# Exendin-4 Reduces Senescence of Inflammation-Induced Periodontal Ligament Stem Cells Through SIRT1/Notch1 Signaling

**DOI:** 10.1155/sci/7639451

**Published:** 2025-11-24

**Authors:** Yunxuan Xu, Jiawen Zheng, Min Liu, Zhuoyu Fu, Ping Wang

**Affiliations:** Department of Stomatology, The First Affiliated Hospital of Chongqing Medical University, Chongqing 400016, China

**Keywords:** exendin-4, inflammatory microenvironment, periodontal ligament stem cells, senescence

## Abstract

Periodontitis is a persistent inflammatory ailment that impacts periodontal tissues. Periodontal ligament stem cells (PDLSCs), also referred to as stem cells, possess advantageous attributes for tissue engineering and regenerative medicine due to their ability to self-renew with multi-directional differentiation potential. Nevertheless, the process of cellular senescence can compromise the restoration and regeneration of tissues, thereby impairing the normal regenerative and reparative functions of the periodontium. Exendin-4 (Ex-4) has protective effects against cellular senescence and apoptosis, but the impact of Ex-4 on inflammation-induced senescence of PDLSCs is unknown. This study used lipopolysaccharide (LPS) to simulate an inflammatory microenvironment, and then assessed the effect of Ex-4 on PDLSC senescence within that environment. Initially, PDLSCs were isolated and characterized and then cultured with LPS or LPS and Ex-4. Results demonstrated that the LPS-induced inflammatory microenvironment produced premature senescence of PDLSCs, which was reversible by treatment with Ex-4. Potential mechanisms underlying the effect were evaluated with regard to senescence-associated molecular pathways, and results demonstrated senescence of PDLSCs to be associated with Sirtuin 1 down-regulation and Notch1 upregulation. Our findings suggest that Ex-4 may mitigate the inflammation-induced senescence of PDLSCs through the SIRT1/Notch1 signaling pathway.

## 1. Introduction

Periodontitis is a global, highly prevalent inflammatory disease, with a steadily rising incidence [1]. Periodontitis is a multifactorial condition, primarily associated with the accumulation of dental plaque [2, 3]. The severe form of periodontitis can cause significant damage to the periodontal ligament and alveolar bone, potentially leading to tooth loss. Periodontitis is also associated with a number of systemic diseases, making the maintenance of periodontal health critical to overall human health [4, 5]. However, conventional techniques such as scaling and root planning only halt the progression of periodontitis by preventing further biofilm development, but cannot restore the missing tissue. The discovery of stem cells within periodontal tissues in 2004 marked the beginning of a new approach to regenerative medicine, stem cell biology, and drug discovery [6]. Periodontal ligament stem cells (PDLSCs) are a group of mesenchymal stem cells (MSCs) derived from the periodontal membrane. PDLSCs have tissue-specific and multidirectional differentiation capabilities, making them a very promising stem cell population for regenerative therapy of periodontal tissues [5, 7].

In a healthy periodontal environment, interactions between host cells and microorganisms elicit a protective immune response that helps maintain periodontal homeostasis [8]. However, senescence of PDLSCs results in a decrease in their ability to proliferate and differentiate [9]. Cellular senescence is a state of permanent cell cycle arrest induced by various forms of stress and certain physiological signals [10, 11]. Inflammation is often recognized as a biomarker of accelerated senescence [12]. Studies [13, 14] have shown that proinflammatory cytokines, such as TNF-α, INF-β, and INF-γ, contribute to epithelial cell senescence through the production of reactive oxygen species (ROS) and activation of the ATM/p53/p21 pathway. Senescence of dental pulp stem cells occurs after repeated stimulation within the inflammatory microenvironment [13, 15]. However, the impact of the inflammatory microenvironment on PDLSCs' senescence has yet to be examined.

Exendin-4 (Ex-4) is a peptide consisting of 39 amino acids that is a full agonist of the glucagon-like peptide 1 (GLP-1) receptor. Previous studies have shown that Ex-4 not only induces insulin secretion [16] but also restores the osteogenic capacity of stem cells. Recently, it has also been shown that Ex-4 also has a role in cellular senescence and apoptosis. Zhou et al. [17] found that Ex-4 protected vascular smooth muscle cells from angiotensin II-induced senescence. Wang et al. [18] found that Ex-4 protected HUVECs from t-BHP-induced apoptosis via PI3K/Akt-Bcl-2-caspase-3 signaling. Oeseburg et al. [19] found that Ex-4 activation of protein kinase A prevented ROS-induced endothelial cell senescence. Furthermore, Ex-4 prevents bile duct cell apoptosis [20] and reverses PC12 cell injury induced by the circRNA, CDR1 as/miR-671/GSK3 β signaling pathway [21]. In recent studies, concerning the impact of Ex-4 on normal PDLSCs, Liang Qianyu et al. found that Ex-4 can enhance the migration ability, chemotaxis, and osteogenic capacity of normal PDLSCs [22, 23]. However, the effects and functions of Ex-4 on the senescence of PDLSCs in the inflammatory microenvironment have not been examined and require further investigation.

The Notch signaling pathway is crucial to cell function and fate. The pathway is influenced by the cellular environment, impacting stem cell differentiation, chemotaxis, reproduction, and aging [24–28]. In terms of senescence, the Notch pathway regulates various mediators of cellular senescence and is significantly connected to senescence-secretory factors [29, 30]. In small cell carcinoma, Notch receptors (Notch1 and Notch2) that are constitutively active have been demonstrated to cause substantial growth arrest. In a standard signaling pathway, the upregulation of the Notch pathway is induced by DLL1, followed by cleavage of the Notch receptor by the γ-secretase complex. The result is the discharge of the Notch intracellular structural domain (NICD) that controls expression of downstream genes [31, 32]. There is increasing evidence that Notch plays a vital role in multiple tooth developmental processes. In periodontal tissues, Notch signaling has been demonstrated to participate in osteogenic differentiation and the induction of osteoclast formation by PDLSCs [33–35]. However, the role of Notch in the senescence of PDLSCs is not fully understood. Decreased expression of silent information regulator 2-related enzyme 1 (SIRT1) is a NAD (nicotinamide adenine dinucleotide)-dependent deacetylase that regulates signaling pathways by modifying target proteins. SIRT1 regulates inflammation, cell senescence, endothelial function, and apoptosis through deacetylation of transcription factors and histones. The level and activity of SIRT1 are reduced in chronic inflammation and aging with oxidative stress [36]. SIRT1 can also regulate DNA repair by promoting the activity of DNA repair enzymes, reducing the accumulation of DNA damage, and protecting cells from DNA damage. In addition, it has been reported that there is an interaction between SIRT1 and the Notch signaling pathway. The Notch1 signaling pathway is the downstream effector pathway of SIRT1, which can affect the occurrence and development of human diseases [37]. However, its role in PDLSCs' senescence is not clear and needs further clarification [38].

In the study, we used lipopolysaccharide (LPS) to simulate the inflammatory environment, observed the senescence of PDLSCs, observed the effect and action of Ex-4 on senescent cells, and explored the related mechanism of action. The results of this study might provide a new reference or basis for understanding PDLSC biology as well as treatment modalities for periodontitis.

## 2. Materials and Methods

### 2.1. Isolation and Culture of Human Periodontal Ligament Cells (hPDLSCs)

PDLSCs were obtained from healthy premolars and molars of young patients under the age of 30 years who had extraction of their healthy premolars and molars for orthodontic purposes or for blocked teeth. The study received approval from the Ethics Committee of Chongqing Medical University. Ethics Number 2022-K64. All patients were informed of the study and signed informed consents. The healthy teeth that were extracted were stored in sterile phosphate-buffered saline (PBS) and kept in an icebox before being taken to the laboratory. The teeth were washed with PBS and immersed in PBS for 5 min. Using a sterile scalpel blade, the periodontal ligament tissue was scraped from the mid-root 1/3 of the surface. The membranes were cut into small pieces, placed into 1.5 mL tubes, and digested with type I collagenase (3 mg/mL) (Sigma–Aldrich, St. Louis, MO, USA) for 30 min at 37°C in a constant temperature water bath. After completion of the digestion, the tissues were placed into T25 cell culture flasks. Then, 5 mL of α-modified minimum essential medium (α-MEM), supplemented with 10% fetal bovine serum (FBS; Gibco, Grand Island, NY, USA) and 1% streptomycin/penicillin, was added. The flasks were then placed in a 37°C CO_2_ incubator, with the medium replaced every 3 days.

### 2.2. Identification of hPDLSCs

We evaluated the colony formation efficiency, differentiation potential, and phenotypic molecular markers (CD146 and STRO-1) of MSCs to identify PDLSCs. Cells used in this study were from the third to the fifth generation.

#### 2.2.1. Colony Formation Assay

Cells were inoculated into 60 mm dishes (800 cells/plate) and cultured in complete medium, which was changed every 2 days. After 14 days, cells were fixed with 4% paraformaldehyde and stained with 0.1% crystal violet. We refer to aggregates of more than 50 cells as colonies.

#### 2.2.2. Oil Red O Staining

The medium for induction of lipogenesis consisted of complete medium containing 1 μmol/L dexamethasone, 10 μmol/L insulin, 200 μmol/L indomethacin, and 0.5 mmol/L 3-isobutyl-1-methylxanthine (IBMX) (Sigma–Aldrich, St. Louis, MO, USA). Cells were inoculated into 60 mm dishes at a density of 1 × 10^6^ cells. After the cells had grown to 80% confluence, the medium was changed to lipid-forming induction medium, with that medium changed every 2 days. After 14 days, the cells were stained for lipid droplets with oil red (Solabio Co. Ltd., Beijing, China).

#### 2.2.3. Alizarin Red Staining

The osteogenic induction medium consisted of complete medium supplemented with 50 μg/mL ascorbic acid, 1 μmol/L dexamethasone, and 3 mmol/L β-glycerophosphate (Sigma–Aldrich, St. Louis, MO, USA). Cells were inoculated into 60 mm dishes at a density of 1 × 10^6^ cells, and after the cells had grown to 80% confluence, the medium was changed to osteogenic induction medium. Osteogenic induction medium was changed every 2 days. After 21 days, the cells were stained with alizarin red (Solabio Co. Ltd., Beijing, China).

#### 2.2.4. Immunofluorescence Staining

For immunofluorescence staining, cells were inoculated into 12-well plates at a density of 2 × 10^4^ cells/well. After stimulation, cells were fixed with 4% paraformaldehyde for 30 min, permeabilized with 0.5% Triton X-100 for 20 min at room temperature, and then incubated with 10% goat serum for 30 min. Cells were incubated overnight at 4°C with the primary antibodies, and then incubated for 1 h at room temperature with the secondary antibody. The following primary antibodies were used: CD146 (1:200; Proteintech Group; Cat. No. 17564-1-AP) and STRO-1 (1:200; Proteintech Group; Cat. No. PE-65184).

Cell nuclei were stained with 4′, 6-diamidino-2-phenylindole (DAPI) for 5 min. Cells were visualized by fluorescence microscopy. The positive rate represents the proportion of stained cells to the total number of cells. The antibodies used in this experiment are listed in Supporting Information 1: Table S1.

### 2.3. Influence of the Inflammatory Microenvironment on PDLSC Senescence

The effect of the inflammatory microenvironment on PDLSC senescence was explored using third- to fifth-generation PDLSCs from the same donor. Cells were all placed at 37°C in a CO_2_ incubator for culture. Control cells (normal group) were cultured in complete medium. Inflammatory group cells (LPS group) were incubated with LPS at concentrations of 0, 0.1, 1, 5, 10, 50, 100, and 150 μg/mL for 1–7 days, with the medium changed every 2 days. Cell activity was measured by CCK8 assays to determine the appropriate LPS concentration. LPS was purchased from Bio-Reagent Company (MCE, Jilin Hongjiu Bio-Tech Co., Ltd.). LPS group cells were cultured in medium containing 10 μg/mL LPS for different times (1–7 days). Apoptotic morphology of cells in the LPS group was observed by Hoechst 33,258 staining to distinguish apoptosis from cell senescence. In subsequent studies, cells in the LPS group were cultured in complete medium containing 10 μg/mL LPS for 6 days, and cells in the normal group were cultured in complete medium for 6 days. Senescence of cells in the LPS group was detected by RNA sequencing, senescence-associated β-galactosidase (SA-β-gal) staining, and cell cycle assessment. Expression of senescence-associated proteins (p53, p-p53, p21, and p16) and DNA damage proteins (p-H2A.X) was assessed by immunoblotting (WB) or quantitative real-time polymerase chain reaction (qRT-PCR) to further evaluate cellular senescence. The effects of cellular senescence were explored by detecting changes in mitochondrial membrane potential (JC-1), ATP, and cellular oxidative stress levels.

Next, we measured cell proliferation, migration, and osteogenic capacity to assess changes in cellular biological function after inflammatory microenvironment-induced senescence. Cell proliferation was assessed using the clone formation assay (Section 2.2.1). Wound healing and infiltration assays were used to detect cell migration. The WB assay and alizarin red staining (Section 2.2.3) were used to detect cellular osteogenesis-related factor (RUNX2) and osteogenic capacity.

#### 2.3.1. CCK-8 Assay

Cells were seeded in 96-well plates (5 × 10^3^ cells/well) and assayed using a CCK-8 kit (ApexBio Technology, Massachusetts, USA) after induction. In each well, 200 μL of medium and 20 μL of CCK-8 assay solution were added, and cells were incubated at 37°C in a CO_2_ light-proof incubator for 2 h. The absorbance (OD) of the samples was measured at 450 nm.

#### 2.3.2. Hoechst 33,258 Staining

Apoptosis-Hoechst Staining Kit (Beyotime Biotechnology Institute, Shanghai, China) was used according to the manufacturer's instructions. PDLSCs were inoculated into 6-well plates (1 × 10^5^ cells/well). Cells were fixed and stained with Hoechst 33,258 staining solution. Three randomly selected fields of view were observed with a fluorescence microscope to visualize nuclear crumpling of the cells and photographed.

#### 2.3.3. SA-β-Gal Staining

Cells were treated in six-well plates at the tested drug concentrations and time, with SA-β-gal staining performed using an SA-β-gal staining kit (Beyotime Biotechnology Institute, Shanghai, China) according to the manufacturer's instructions. First, 1 mL of β-galactosidase staining fixative was added to each well at room temperature for 15 min, rinsed three times with PBS, the staining solution was added, and the 6-well plate was placed in a 37°C CO_2_-free incubator overnight. The next day, samples were photographed using an inverted microscope with a digital camera. Senescent cells appeared blue by light microscopy. Three fields of view (at least 100 cells per field of view) were randomly selected to determine the degree of senescence:

  $$\begin{array}{c} \left(\beta -\text{gal positive cells}/\text{total number of cells in the field of view}\right)\times 100\%. \end{array}$$

#### 2.3.4. Mitochondrial Assessment

Staining was performed in 6-well plates using a Mitochondrial Membrane Potential Detection Kit (JC-1) (Beyotime Biotechnology Institute, Shanghai, China) according to the manufacturer's instructions, observed and photographed using a fluorescence microscope. Changes in mitochondrial membrane potential were detected by fluorescence color shifts, and the number of positive mitochondrial membrane potential injuries was calculated using ImageJ. Damage rate (%) = ratio of stained cells to total number of cells.

ATP, as a major energy molecule, plays an important role in a variety of physiological and pathological processes within cells, with changes in ATP levels affecting cell function. Using the Enhanced ATP Assay Kit (Beyotime Biotechnology Institute, Shanghai, China), wall-attached cells were lysed with the lysis solution provided in the kit, and experiments were performed according to the steps provided in the kit, with final detection by chemiluminescence.

#### 2.3.5. Measurement of Oxidative Stress Levels

Intracellular ROS levels were measured using an ROS assay kit according to the manufacturer's instructions. Briefly, after completion of cell stimulation, cells were treated with 10 μmol/L DCFH-DA and incubated at 37°C for 20 min. ROS generation was assessed by analysis of fluorescence intensity. To determine MDA levels and SOD activity, cell supernatants were collected at the end of stimulation and assessed by western blot analysis of immune precipitates (IPs) of cell lysates (Beyotime Biotechnology Institute, Shanghai, China). MDA levels and SOD activity were determined using kits according to the manufacturer's instructions. The kits used in these experiments were manufactured by Beyotime Institute of Biotechnology Co., Ltd. (Shanghai, China).

#### 2.3.6. Transwell Migration Assay

PDLSCs of generation 3–5 were inoculated into 6-well plates (1 × 10^5^ cells/well). After stimulation was complete, PDLSCs were added to α-MEM in the upper chamber at a density of 2 × 10^4^ cells/well, with normal medium added to the lower chamber. After 24 h of incubation, cells migrating to the sub-membrane surface were fixed and stained with 1% crystal violet. Samples were photographed using a digital camera and an inverted microscope. The number of migrated cells was calculated using ImageJ.

#### 2.3.7. Wound Healing Assay

PDLSCs of generation 3–5 were inoculated into 6-well plates (1 × 10^5^ cells/well). Upon completion of stimulation, wound healing assays were performed using a 200 µL pipette tip. After scratching the cell monolayer, photographs were taken at standard time intervals (0, 24, 48, and 72 h). To determine the wound healing rate, calculations were based on the initial scratch area and the change in scratch area over time. The formula for calculating the wound healing rate is as follows:

  $$\begin{array}{c} \left(\text{initial scratch area}-\text{scratch area at a given time}\right)/\text{initial scratch area}\times 100. \end{array}$$

#### 2.3.8. Cell Cycle Assay

A cell cycle detection kit (Lianke Bio) was used. Cells were digested with trypsin (Beyotime Institute of Biotechnology, Shanghai, China), and 2 × 10^5^ −1 × 10^6^ cells were collected by centrifugation. The supernatants were discarded. The cells were washed with PBS, a mixture of 1 mL of DNA Staining Solution and 10 μL of Permeabilization Solution was added, and mixed by vortexing for 5−10 s. The cells were incubated for 30 min at room temperature in the absence of light, and analyzed by flow cytometry (CytoFLEX Beckman Coulter). The percentage of cells in various stages of the cell cycle was calculated by CModFit LT 5.0 software analysis.

#### 2.3.9. RNA Sequencing

RNA was extracted from various PDLSC groups using RNAiso Plus (Takara, Japan). High-throughput sequencing was used to eliminate unqualified sequences (including sequencing junctions, low-quality sequences, etc.) from the raw data with Cutadapt, yielding valid data (Clean Data). Hisat2 was used for comparison to the reference genome, whereupon the transcripts were reconstructed and expression levels calculated for each gene in every sample using StringTie in accordance with the Hisat2 results. The resulting expression levels were then analyzed. The analysis of gene expression levels focused on protein-coding genes annotated within the genome, specifically mRNA. The expression of genes is tallied to evaluate the relationships among gene expression characteristics and differentially expressed genes within and between groups of samples.

#### 2.3.10. Western Blotting

Proteins were extracted with RIPA intensifier (including inhibitors for proteases and phosphatases) according to the protocol provided by the manufacturer (Beyotime Institute of Biotechnology, Shanghai, China). The concentration of protein in cellular lysates was analyzed by the M5 BCA Protein Assay Kit (Mei5Bio, Beijing, China). The samples were boiled for 10 min in sample buffer at a 4:1 ratio. Protein samples were separated via polyacrylamide electrophoresis and transferred onto polyvinylidene fluoride (PVDF) membranes (Millipore, city, state, USA). The membranes were sealed and incubated in 5% skim milk and 0.2% Tween in tris-buffered saline (TBS) for 2 h at room temperature. The membranes were incubated in the corresponding primary antibodies overnight at 4°C in the refrigerator. The following primary antibodies were used: p53 (1:1000; Proteintech Group; Cat. No. 10442-1-AP), p-p53 (1:1000; Proteintech Group; Cat. No. R8961-1-AP), p21 (1:1000; Proteintech Group; Cat. No. 10355-1-AP), p16 (1:1000; Proteintech Group; Cat. No. 10883-1-AP), and p-H2A.X (1:1000; Abcam; Cat. No. ab131382). The following day, the membranes were incubated with secondary antibody (dissolved in TBS-Tween 20) for 2 h at room temperature. Proteins were detected using the MCE ultrasensitive ECL chemiluminescence kit in accordance with the manufacturer's protocol. The gray value of the target bands was calculated using Image J software (version v1.8.0; National Institutes of Health). Goat anti-rabbit IgG horseradish peroxidase (1:5000, Affinity Biosciences, Jiangsu, China) was utilized as the secondary antibody. The antibodies that were employed in this experiment are listed in Supporting Information 1: Table S1.

#### 2.3.11. qRT-PCR

PDLSCs were cultured as described above. On day 7, total RNA was extracted using a Rapid RNA Kit (Accurate Bio). An Evo M-MLV Reverse Transcription Premix Tracer Kit (Accurate Bio) was used to reverse transcribe total RNA into cDNA. qRT-PCR was performed using SYBR Green qPCR Master Mix (No ROX) (MCE, Jilin Hongjiu Biotechnology Co., Ltd.) using BIO-RAD CFX Manager Connect PCR Detection System software (Bio-Rad). qRT-PCR was used to analyze the expression of p21 and p53 genes, with GAPDH used to determine their relative expression. The expression levels of p21 and p53 genes were analyzed by qRT-PCR and normalized. The primers used in this study are listed in Supporting Information 2: Table S2, below.

### 2.4. Effect of Ex-4 Treatment on Senescent PDLSCs

Ex-4 was purchased from Bio-reagent Company (MCE, Jilin Hongjiu Biotechnology Co., Ltd.). Normal PDLSCs and senescent PDLSCs were treated with 0, 5, 10, 20, 50, 100, and 150 nmol/L Ex-4 for 48 h. The appropriate concentration of Ex-4 was determined by the CCK-8 assay (Section 2.3.1). Based on the results, we selected 10 nmol/L Ex-4, which was incubated with senescent PDLSCs for 48 h (LPS + Ex-4 group) and with normal PDLSCs for 48 h (Ex-4 group). PDLSCs in the control group (normal group) and in the inflammatory group (LPS group) received a similar volume of PBS. The inflammatory microenvironment was assessed by SA-β-gal staining (Section 2.3.3) to assess inflammatory microenvironment-induced cellular senescence in response to Ex-4 treatment. Similarly, the expression of senescence-associated proteins was detected using WB (Section 2.3.10) or qRT-PCR (Section 2.3.11). In addition, we assessed the expression of the senescence-associated secretory phenotype (SASP: IL-1β, IL-6, and IL-8) genes using an enzyme-linked immunosorbent assay (ELISA). Subsequently, we examined the migratory and proliferative capacities of the cells (Section 2.3). We examined osteogenic capacity by alkaline phosphatase (ALP), alizarin red staining (Section 2.2.3), and WB assays (Section 2.3.10) to determine changes in cellular biologic function.

#### 2.4.1. ELISA

Cells were inoculated into 12-well plates at a density of 1 × 10^5^ cells per well. The supernatants of each group were extracted after the completion of incubation. Senescence-associated secretory phenotype proteins (SASP: IL-1β, IL-6, and IL-8) in supernatants were assessed with Human Interleukin 1β (IL-1β), Human Interleukin 6 (IL-6), and Human Interleukin 8 (IL-8) ELISA kits (MLbio, China) according to the manufacturer's instructions. The absorbance of the samples was measured at 450 nm. Optical density (OD) values were compared to a standard curve to determine the concentrations of IL-1β, IL-6, and IL-8.

#### 2.4.2. ALP Staining Assay and ALP Activity Measurement

PDLSCs were inoculated into 6-well plates (1 × 10^5^ cells/well), and when cell confluence reached 80%, osteogenic medium was added and cells were cultured for 7 days, when ALP staining and ALP activity assays were performed. For the ALP staining assay, an ALP color development kit (Beyotime Biotechnology Institute, Shanghai, China) was used according to the manufacturer's instructions. The ALP activity of the cells was determined according to the manufacturer's instructions (Beyotime Biotechnology Institute, Shanghai, China) and normalized to the protein concentration. The absorbance of each well was measured at 520 nm.

### 2.5. Effect of Ex-4 on the Growth of Xenografts in a Nude Mouse Model

This study utilized 8-week-old male nude mice. The nude mice were purchased from Hunan Slake Jinda Laboratory Animal Co., Ltd. and were kept in the Animal Management Center of Chongqing Medical University. Animal experiments were performed in accordance with national and institutional guidelines for the care and use of animals, and were approved by the Animal Ethics Committee of Chongqing Medical University (Ethics Number 20230296). The cells were divided into the normal group, the Ex-4 group, the LPS group, and the LPS + Ex-4 group in vitro, and osteogenic induction culture was performed for 21 days. Twelve nude mice were divided into four groups (three nude mice in each group): normal group, Ex-4 group, LPS group, and LPS + Ex-4 group. After centrifugation, cells from each group were collected and diluted with 180 μL PBS, respectively. Then, cells from each group were mixed with 20 μL of Matrigel (Corning), respectively, and subcutaneously transplanted into the groin of nude mice. The cell count was 2.0 × 10^7^/mouse. First, anesthetize the nude mice with isoflurane and slowly inject 200 μL of the cell suspension into the groin. Then the graft was removed 1 month later. The surgeries were performed under general anesthesia by intraperitoneal injection of 1% phenobarbital sodium. Grafts were removed and fixed with 4% paraformaldehyde, decalcified with buffered 10% ethylenediaminetetraacetic acid (EDTA, pH 8.0), embedded in paraffin, and cut into 5 mm sections.

To observe the effect of Ex-4 on osteogenesis and the proliferation of PDLSCs in vivo, WB (Section 2.3.10), micro-CT, and immunofluorescence (Section 2.2.4). Immunohistochemical staining was used to detect the proliferation-related proteins (PCNA, Ki67), senescence-related protein p21, and osteogenic factors (RUNX2, OPG). The proteins used in the WB experiment were extracted from the xenograft tissues. This analysis was performed on samples transplanted for 4 weeks, with cell proliferation and osteogenic potential assessed. The antibodies used in this experiment are listed in Supporting Information 1: Table S1.

#### 2.5.1. Immunohistochemistry

Samples were fixed in 4% paraformaldehyde for 10 min and washed with distilled water. Cells were treated with 3% H_2_O_2_ for 15 min at room temperature and blocked with normal goat serum for 15 min. Primary antibodies were then added and incubated at 4°C overnight. The following primary antibodies were used: p21 (1:200; Proteintech Group; Cat. No. 10355-1-AP) and OPG (1:200; Huabio Biosciences; Cat. No. R1608-4). The next day, sections were incubated with IgG-HRP secondary antibody. The sections were visualized with a microscope and photographed. The antibodies used in this experiment are listed in Supporting Information 1: Table S1.

### 2.6. Role of SIRT1/Notch1 in Ex-4 Mediated Delay of PDLSC senescence

Before and after Ex-4 treatment of senescent PDLSCs, we detected alterations in SIRT1 and Notch signaling pathway-related proteins (Notch1, NICD, DLL1, Hey1, Hes1) by WB (Section 2.3.10), qRT-PCR (Section 2.3.11), or immunofluorescence (Section 2.2.4) to investigate the involvement of SIRT1/Notch1 in the senescence of PDLSCs with Ex-4.

To evaluate the role of Ex-4 in the delay of the senescence of PDLSCs (by regulating SIRT1/Notch1), we added a SIRT1 inhibitor (EX-527). The experimental groups were: normal group, Ex-4 group, Ex-4 + EX-527 group, LPS group, LPS + Ex-4 group, and LPS + Ex-4 + EX-527 group. Changes in Notch signaling pathway-related proteins (Notch1, NICD, Hes1), SIRT1, and aging-related proteins (p53, p21) were detected by WB (Section 2.3.10). A Notch signaling pathway inhibitor (DAPT) was then added. The experimental groups were: normal group, Ex-4 group, Ex-4 + DAPT group, LPS group, LPS + Ex-4 group, and LPS + Ex-4 + DAPT group. Changes in Notch signaling pathway-related proteins (Notch1, NICD, Hes1), SIRT1, and aging-related proteins (p53, p21) were detected by WB (Section 2.3.10) to evaluate the relationship between SIRT1 and Notch1. To further analyze the relationship between SIRT1 and Notch1, coimmunoprecipitation analysis was performed. The antibodies used in this experiment are listed in Supporting Information 1: Table S1.

#### 2.6.1. Immunoprecipitation Analysis

An immunoprecipitation kit, protein A + G magnetic bead assay (Beyotime Institute of Biotechnology, Shanghai, China), was used according to the manufacturer's instructions. Cells were lysed, centrifuged at 14,000 × *g* for 3–5 min at 4°C, and supernatants removed. The antibody was diluted with TBS at the dilution ratio and set aside on ice. After washing, the protein A + G beads were magnetically separated, the supernatants were aspirated, and 500 μL of antibody working solution was added and incubated at room temperature for 1 h. Then, 500 μL of TBS was added and the protein A + G magnetic beads resuspended by blowing, then placed on a magnetic rack to separate the supernatant. This procedure was repeated three times. The magnetic beads were resuspended in TBS to the initial volume. Samples were incubated with antibody-conjugated magnetic beads overnight. After incubation, samples were placed on a magnetic rack and separated for 10 s to remove supernatants. This procedure was repeated three times with inhibitor-containing lysate, followed by elution. For every 20 μL of original magnetic bead volume, 100 μL of SDS–PAGE sample loading buffer (1x) was added and then heated at 95°C for 5 min. Samples were placed on a magnetic rack to separate for 10 s, and the supernatants were used for western blot analysis (Section 2.3.10).

### 2.7. Statistical Analysis

PDLSCs from the same donor were utilized in each experiment and repeated at least three times. Statistical analysis was conducted using GraphPad Prism 9 software. Results are presented as means ± SD. Differences between groups were assessed for statistical significance using *t*-tests. For multiple comparisons, ANOVA was employed. Each experiment was repeated with PDLSCs from at least three distinct donors. Differences were deemed statistically significant at *p* < 0.05 (*⁣*^*∗*^), *p* < 0.01 (*⁣*^*∗∗*^), *p* < 0.001 (*⁣*^*∗∗∗*^), *p* < 0.0001 (*⁣*^*∗∗∗∗*^), or ns (not significant).

## 3. Results

### 3.1. PDLSC Morphology and Characterization

**Figure 1:**
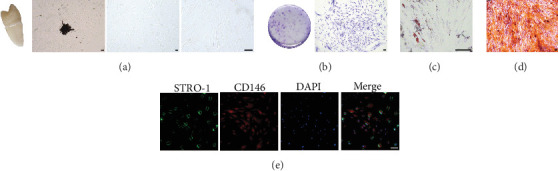
Culture and characterization of human PDLSCs. (A) Isolation and culture of human PDLSCs. Primary cells grew and multiplied around the tissue mass, and the cells grew in a spindle shape. Scale bar = 100 μm. (B) After 14 days of culture, colonies formed by PDLSCs were stained with crystal violet to show the proliferation of PDLSCs. Scale bar = 100 μm. (C) Staining of lipid droplets with oil red O staining after induction of cell lipogenesis, showing the ability of cells to differentiate into adipocytes. Scale bar = 100 μm. (D) Alizarin red staining of calcified nodules after osteogenic induction, showing the ability of PDLSCs to differentiate into osteoblasts. Scale bar = 100 μm. (E) Human PDLSCs expressing STRO-1 (green) and CD146 (red). Scale bar = 100 μm.

Primary cells migrated from the tissue periphery and underwent growth and proliferation (Figure 1A). PDLSCs exhibited a spindle or fibroblast-like morphology and grew in a regular and directional manner (Figure 1A). After 14 days in culture, isolated cells formed colonies (Figure 1B). Oil red O staining after 14 days of culture confirmed the differentiation of PDLSCs into lipid-forming cells (Figure 1C). Alizarin red staining after 21 days of osteogenic induction confirmed PDLSCs differentiated into osteoblasts, with calcified nodules clearly visible (Figure 1D). PDLSCs demonstrated expression of MSC phenotypic molecular markers, CD146 and STRO-1, as judged by fluorescence staining (Figure 1E).

### 3.2. An Inflammatory Environment Induces Senescence of PDLSCs

**Figure 2:**
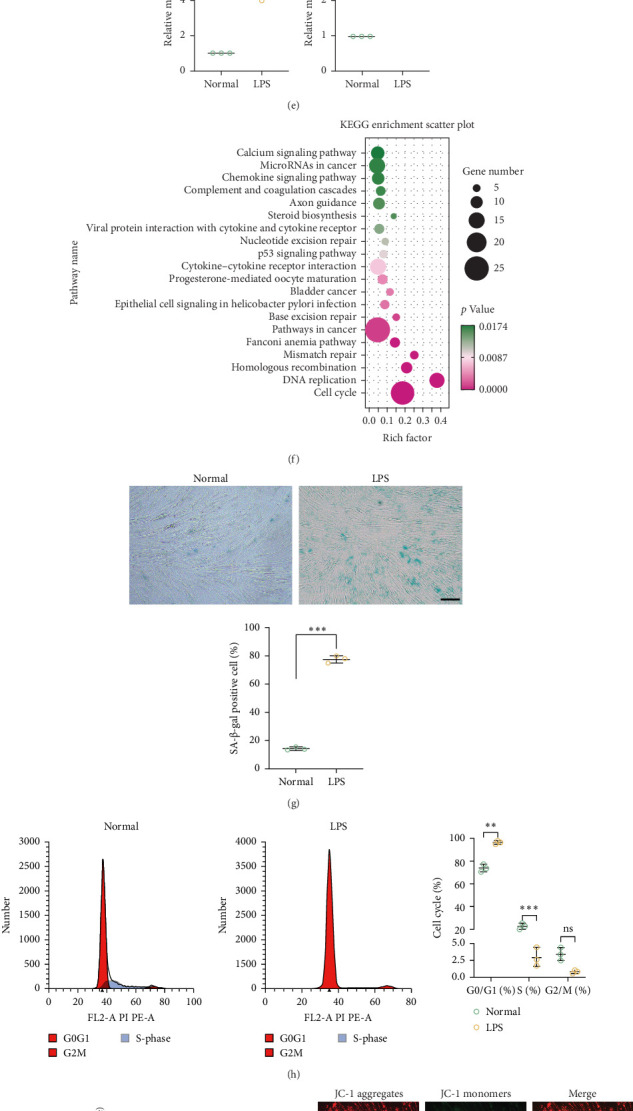
Inflammatory microenvironment-induced senescence in human PDLSCs. Experimental groups: control group (normal group), inflammation group (LPS group). The LPS group was cultured with different concentrations of LPS, and the normal group was cultured with complete medium. First, the effect of LPS on the proliferation of PDLSCs was investigated (A–C). (A) Growth curves of PDLSCs after 6 days of intervention with LPS at concentrations of 0.1–150 μg/mL (B). The growth curves of PDLSCs treated with LPS at a concentration of 10 μg/mL for 1–7 days. Data are expressed as the mean (SD) of triplicate samples from the experiment. (C) Apoptosis was observed by Hoechst 33,258 staining after intervention with LPS at a concentration of 10 μg/mL for 1–7 days; apoptosis appeared on day 7, and apoptotic nuclei appeared in the form of clumps (red arrows). Scale bar = 100 μm. Enlarged image scale bar = 20 μm. (D–F) 10 μg/mL LPS was used to continuously culture cells for 6 days for subsequent experiments. The sequencing results were analyzed by heatmap and KEGG enrichment. The expression levels of senescence genes (p21 and p53) in the normal and the LPS group were verified by qRT-PCR. Then, the phenotypic changes of senescence in the normal and LPS groups were observed. (G) Representative images of SA-β-gal-positive cells (blue) in PDLSCs in the normal and LPS groups and quantification of the percentage of SA-β-gal-positive cells in each group. The percentage of SA-β-gal-positive cells within the LPS group was significantly increased. Scale bar = 100 μm. (H) Cell cycle analysis of the normal and LPS groups by flow cytometry. (I) Western Blot to detect the expression of cellular senescence-associated proteins (p53, p-p53, p21, and p16) and DNA damage proteins (p-H2A.X) in the normal and LPS groups. (J) Representative images of MMP in cells of the normal and LPS groups after JC-1 staining. Scale bar = 100 μm. (K) Changes in intracellular mitochondria in the normal and LPS groups were assessed by detecting ATP levels. (L) Intracellular reactive oxygen species (ROS) levels were assessed by DCFH-DA fluorimetry in the normal and LPS groups. ROS levels were significantly increased in the LPS group. Scale bar = 100 μm. (M) Detection of cellular oxidative stress levels in the normal and LPS groups by measuring MDA levels and SOD activity. Data are presented as mean ± standard deviation (*n* = 3 independent observations). *⁣*^*∗*^*p* < 0.05; *⁣*^*∗∗*^*p* < 0.01; *⁣*^*∗∗∗*^*p* < 0.001; ns, not significant.

In this study, we investigated whether an inflammatory microenvironment induces senescence in PDLSCs. To simulate the inflammatory conditions associated with periodontitis, LPS was used to establish an in vitro inflammatory microenvironment. Initial experiments showed that after 6 days of culture, cell proliferation was enhanced at LPS concentrations up to 5 µg/mL, whereas suppression of cell growth was observed at concentrations exceeding 10 µg/mL (Figure 2A). To further characterize the time-dependent effect, PDLSCs were treated with 10 µg/mL LPS for up to 7 days. As illustrated in Figure 2B, LPS promoted proliferation during the first 3 days of treatment but significantly inhibited cell growth from day 5 onward. Moreover, cells treated with LPS (10 µg/mL) showed significant apoptosis on day 7 (Figure 2C). We therefore chose to stimulate cells with 10 µg/mL LPS for 6 days for subsequent experiments, with sequencing results demonstrating a significant rise in p53 and p21 (Figure 2D,E). By KEGG enrichment analysis of the sequencing results (Figure 2F), LPS induced an inflammatory environment that was closely related to cellular senescence. PDLSCs that were exposed to 10 µg/mL LPS for 6 days exhibited typical features of senescence including; increased SA-β-gal staining (Figure 2G), a significant cell cycle arrest in the G0/G1 phase (Figure 2H), significantly heightened levels of senescence-associated proteins (p53, p-p53, p21, and p16), and the DNA damage protein (p-H2A.X) (Figure 2I). Altered mitochondrial membrane potential and oxidative stress are characteristics of aging. The inflammatory microenvironment led to significant changes in the mitochondrial membrane potential of PDLSCs, as well as heightened levels of oxidative stress (Figure 2).

**Figure 3:**
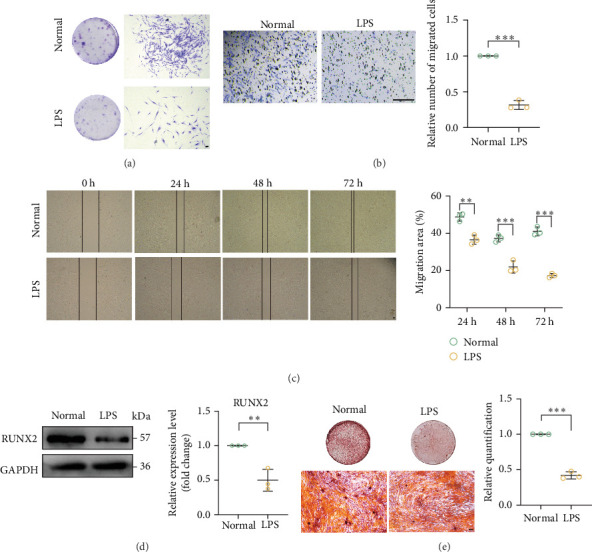
The dysfunction of human PDLSCs may result from an inflammatory microenvironment. (A) The proliferation ability of cells in the normal and LPS groups was detected by the plate colony formation assay. Scale bar = 100 μm. (B, C) The ability of cell migration in the normal and LPS groups was assessed by wound healing and permeabilization assays. Scale bar = 100 μm. (D) Western Blot detected the expression of osteogenesis-related factor (RUNX2) in the cells of normal and LPS groups, and the expression of RUNX2 was decreased in senescent cells. (E) Osteogenic capacity of cells in the normal and LPS groups was assessed by alizarin red staining and quantification of mineralized nodules. The LPS group had decreased osteogenic capacity. Scale bar = 100 μm. Data are presented as mean ± standard deviation (*n* = 3 independent observations). *⁣*^*∗∗*^*p* < 0.01; *⁣*^*∗∗∗*^*p* < 0.001.

Furthermore, the inflammatory microenvironment decreased cell proliferation (Figure 3A), cell migration (Figure 3B,C), and osteogenic capacity (Figure 3D,E) of PDLSCs. Based on these findings, we concluded that an inflammatory microenvironment induced premature senescence of PDLSCs.

### 3.3. Ex-4 Delays Senescence of PDLSCs in the Inflammatory Microenvironment

**Figure 4:**
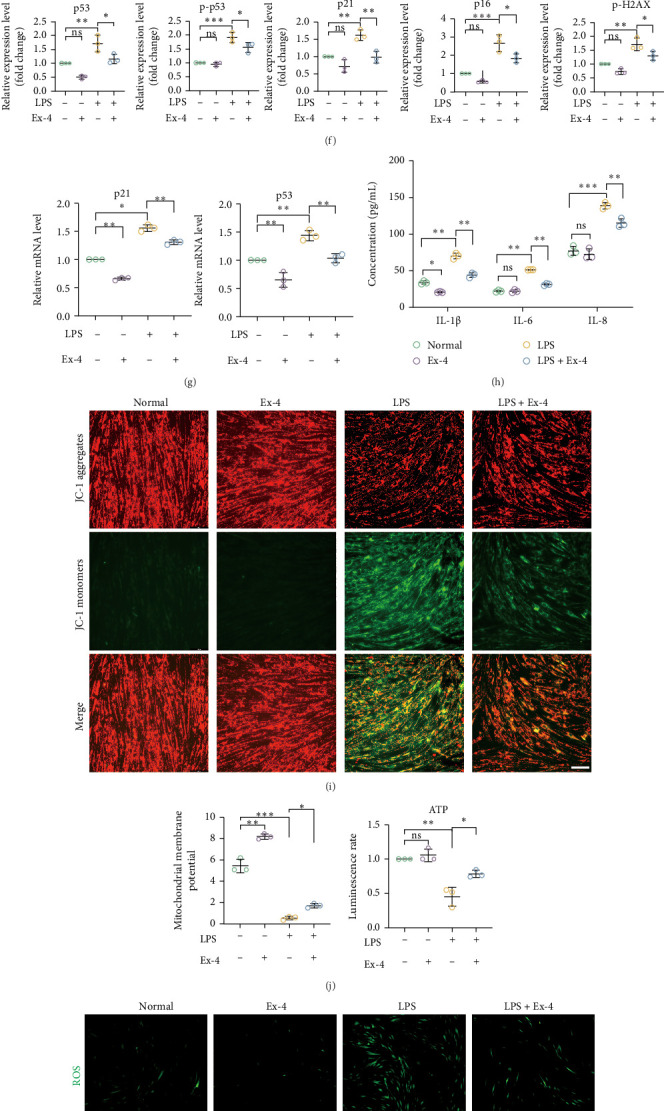
Ex-4 ameliorates inflammatory microenvironment-induced senescence of human PDLSCs. The appropriate concentration of Ex-4 was first determined. Normal and senescent human PDLSCs were treated with 0, 5, 10, 20, 50, 100, and 150 nmol/L Ex-4 for 48 h, and the effects of Ex-4 and/or LPS on the proliferation of PDLSCs were detected by CCK8 (A, B). (A) Growth curves of human PDLSCs after 48 h of intervention at Ex-4 concentrations of 5–100 nmol/L. (B) Growth curves of human PDLSCs after 6 days of LPS (10 μg/mL) treatment and 48 h of Ex-4 (10 nmol/L) treatment; data are expressed as the mean (SD) of three samples in the experiment. Subsequent experiments were grouped into the following groups: normal group (equal volume of PBS treatment), Ex-4 group (10 nmol/L), LPS group (10 μg/mL LPS + equal volume of PBS treatment), and LPS + Ex-4 group (10 μg/mL LPS + 10 nmol/L Ex-4). (C, D) The role of Ex-4 in delaying senescence of human PDLSCs was assessed by SA-β-gal staining. Representative images of cells in each group and quantitative statistics of the percentage of SA-β-gal positive cells. Scale bar = 100 μm. (E, F) Western Blot detected the expression of cellular senescence-related proteins (p53, p-p53, p21, and p16) and DNA damage proteins (p-H2A.X) in the normal, Ex-4, LPS, and LPS + Ex-4 groups. (G) The expression levels of senescence genes (p21 and p53) in the normal, Ex-4, LPS and LPS + Ex-4 groups were assessed by qRT-PCR. (H) Detection of SASP (IL-1β, IL-6, and IL-8) in the culture medium supernatant of normal, Ex-4, LPS, and LPS + Ex-4 groups by ELISA. (I) Representative images of MMP in cells of normal, Ex-4, LPS and LPS + Ex-4 groups were observed by JC-1 staining. Scale bar = 100 μm. (J) Intracellular mitochondrial changes were assessed by measuring ATP in the normal, Ex-4, LPS, and LPS + Ex-4 groups. (K) Intracellular reactive oxygen species (ROS) levels were assessed by DCFH-DA fluorometric assay in normal, Ex-4, LPS, and LPS + Ex-4 groups. Scale bar = 100 μm. (L) Detection of cellular oxidative stress levels in normal, Ex-4, LPS, and LPS + Ex-4 groups by measuring MDA levels and SOD activity. Data are presented as mean ± standard deviation (*n* = 3 independent observations). *⁣*^*∗*^*p* < 0.05; *⁣*^*∗∗*^*p* < 0.01; *⁣*^*∗∗∗*^*p* < 0.001; ns, not significant.

PDLSCs in their normal state were treated with varying concentrations of Ex-4 (0−150 nmol/L) for 48 h. Results showed that a 10 nmol/L concentration of Ex-4 promoted cell proliferation, that a 50 nmol/L concentration of Ex-4 produced the greatest proliferation rate, while a 100 nmol/L concentration of Ex-4 inhibited cell proliferation (Figure 4A). Treatment of senescent PDLSCs with varying concentrations of Ex-4 (0−150 nmol/L) for 48 h demonstrated that an Ex-4 concentration of 0−20 nmol/L increased cell proliferative capacity (Figure 4B). Results suggested that Ex-4 enhanced cell viability in an inflammatory environment. Therefore, this study used Ex-4 at a concentration of 10 nmol/L in subsequent experiments with an equal volume of PBS as a placebo. After the addition of 10 nmol/L Ex-4 to senescent cells, the positive rate of SA-β-gal staining was significantly reduced (Figure 4C,D). By western blot analysis and qRT-PCR, Ex-4 was shown to inhibit senescence-induced DNA damage and to reduce the expression of senescence markers p16, p21, p-p53, and p53 (Figure 4E–G). In addition, the levels of senescence-associated secretory phenotypes (IL-1β, IL-6, and IL-8) were significantly decreased (Figure 4H). Changes in mitochondrial membrane potential were delayed after Ex-4 treatment (Figure 4I,J). Production of ROS was also somewhat inhibited, which may reduce the level of oxidative stress by enhancing the endogenous antioxidant system (Figure 4K,L).

**Figure 5:**
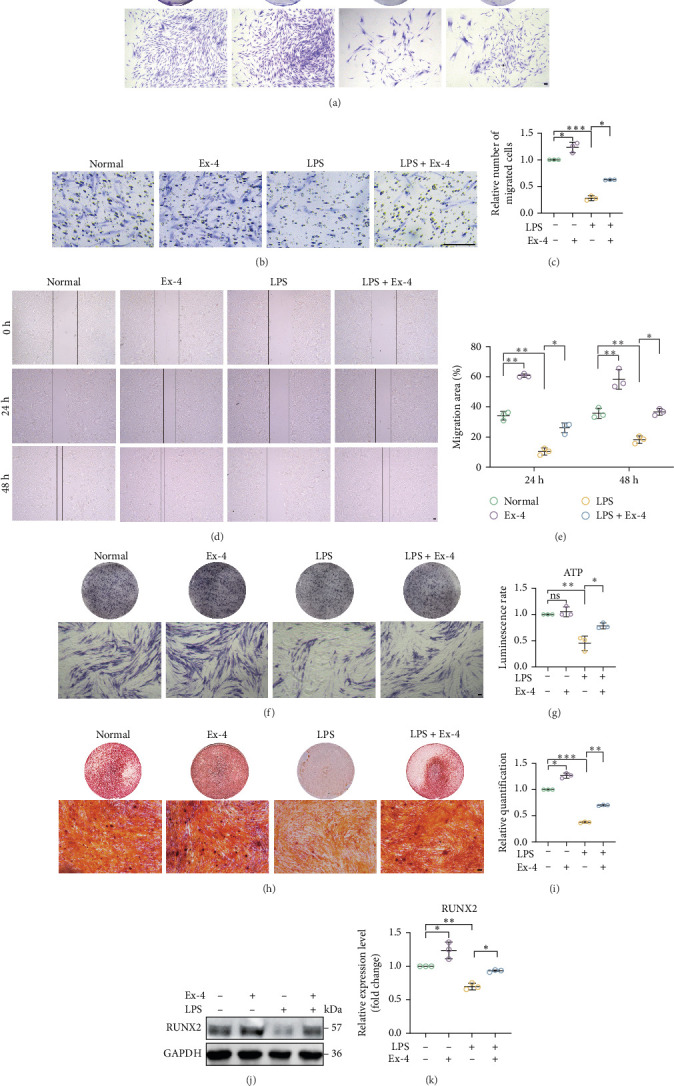
Ex-4 attenuates inflammatory microenvironment-induced dysfunction in senescent human PDLSCs. (A) The effect of Ex-4 on the proliferative capacity of senescent PDLSCs was assessed by plate colony formation assay. Scale bar = 100 μm. (B–E) The ability of cell migration in normal, Ex-4, LPS, and LPS + Ex-4 groups was assessed by wound healing and permeabilization assays. Scale bar = 100 μm. (F, G) ALP staining/activity assay of cells in normal, Ex-4, LPS, and LPS + Ex-4 groups. Scale bar = 100 μm. (H, I) Osteogenic differentiation capacity of cells in normal, Ex-4, LPS, and LPS + Ex-4 groups was assessed by alizarin red staining and quantification of mineralized nodules. Scale bar = 100 μm. (J, K) Western blot detection of RUNX2 expression in normal, Ex-4, LPS, and LPS + Ex-4 groups. Data are presented as mean ± standard deviation (*n* = 3 independent observations). *⁣*^*∗*^*p* < 0.05; *⁣*^*∗∗*^*p* < 0.01; *⁣*^*∗∗∗*^*p* < 0.001; ns, not significant.

Because PDLSC biological function deteriorates with age, we investigated the effect of Ex-4 on the biological characteristics of senescent PDLSCs induced by an inflammatory microenvironment. The plate colony formation assay (Figure 5A) demonstrated that Ex-4 restores the proliferation of senescent PDLSCs induced by an inflammatory microenvironment. Ex-4 treatment improved the migration of senescent PDLSCs as demonstrated by both transwell and wound healing assays (Figure 5B–E). In addition, incubation of senescent PDLSCs with Ex-4 improved cellular osteogenic differentiation (Figure 5F–K). Results demonstrated that Ex-4 improved the biological function of senescent PDLSCs.

### 3.4. Ex-4-Treated Senescent PDLSCs Exhibit Enhanced Growth and Osteogenesis In Vivo

**Figure 6:**
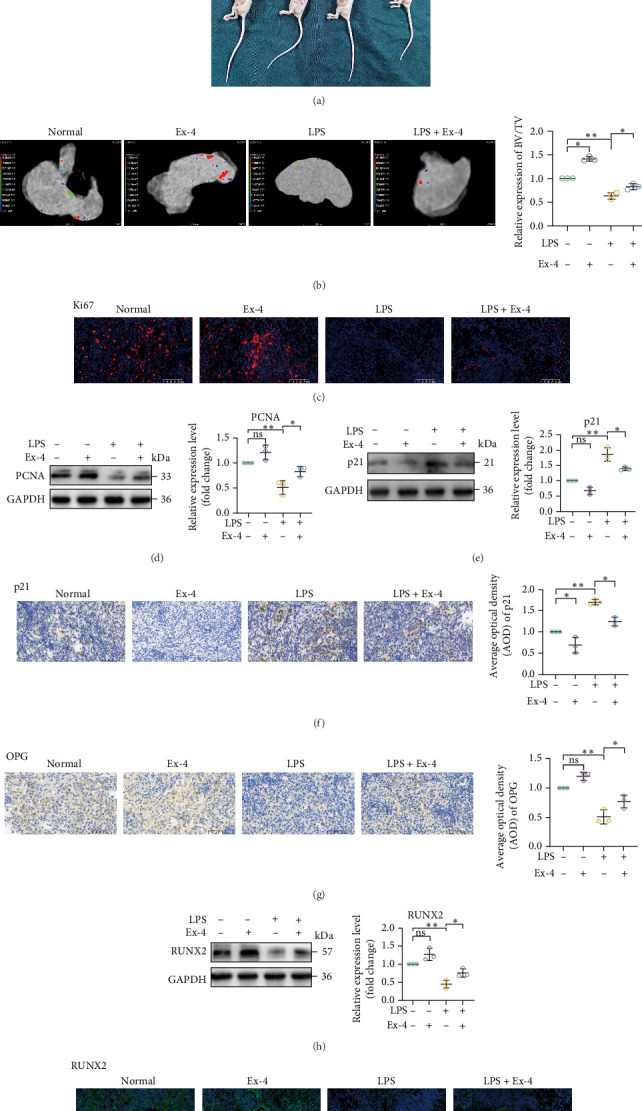
Ex-4 promotes the expression of osteogenic differentiation factors in human PDLSCs in vivo. Experimental groups: normal group (equal volume of PBS treatment), Ex-4 group (10 nmol/L), LPS group (10 μg/mL LPS + equal volume of PBS treatment), and LPS + Ex-4 group (10 μg/mL LPS + 10 nmol/L Ex-4). (A) After osteogenic induction in vitro, cells from each group were collected and injected subcutaneously into nude mice, and PDLSC grafts were removed 4 weeks later. (B) Microscopic CT images and relative expression of graft volume fraction (BV/TV) in the normal, Ex-4, LPS, and LPS + Ex-4 groups. (C) The proliferative capacity of the grafts in the normal, Ex-4, LPS, and LPS + Ex-4 groups was assessed by immunofluorescence staining for Ki67. Scale bar = 100 μm. (D) Western Blot detection of PCNA expression in grafts from normal, Ex-4, LPS, and LPS + Ex-4 groups. (E) Western Blot detection of p21 expression in grafts from normal, Ex-4, LPS and LPS + Ex-4 groups. (F) Immunohistochemical staining plots and statistical analysis of p21 in grafts from normal, Ex-4, LPS and LPS + Ex-4 groups. Scale bar = 100 μm. (G) The osteogenic capacity of the grafts in the normal, Ex-4, LPS, and LPS + Ex-4 groups was assessed by immunohistochemical staining of OPG. Scale bar = 100 μm. (H) Western blot detection of RUNX2 expression levels in grafts from normal, Ex-4, LPS, and LPS + Ex-4 groups. (I) The osteogenic capacity of the grafts in the normal, Ex-4, LPS and LPS + Ex-4 groups was evaluated by immunofluorescence staining with RUNX2. Scale bar = 100 μm. Data are presented as mean ± standard deviation (*n* = 3 independent observations). *⁣*^*∗*^*p* < 0.05; *⁣*^*∗∗*^*p* < 0.01; ns, not significant.

In vitro, significant differences were observed in the proliferation rates between control and senescent cells under inflammatory conditions. Importantly, Ex-4 treatment effectively rescued the impaired proliferation and restored the osteogenic capacity of these senescent cells. To evaluate whether these beneficial effects could be translated in vivo, we employed a nude mouse xenograft model. Following a 4-week transplantation period, the PDLSC grafts were harvested for analysis (Figure 6A). Through micro-CT analysis, we quantified the relative volume of mineralized bone tissue in allogeneic grafts using bone volume fraction (BV/TV). The results showed that after treatment with Ex-4, the relative volume of mineralized bone tissue within the group increased significantly (Figure 6B). The senescent cell group had significantly fewer proliferating cells than the control cell group, while the Ex-4-treated group had significantly more proliferating cells than the senescent cell group (Figure 6C,D). These results suggested that the growth of senescent PDLSCs was impeded in an inflammatory microenvironment. The senescence marker, p21, was found to be decreased in the Ex-4-treated senescent group by western blot analysis (Figure 6E) and immunohistochemical staining (Figure 6F). Immunohistochemical detection of osteoprotegerin (OPG) and immunofluorescence staining of RUNX2 showed that Ex-4 enhanced osteogenic potential (Figure 6G–I). In conclusion, these findings suggested that Ex-4-treated PDLSCs were capable of enhanced osteogenic differentiation both in vitro and in vivo.

### 3.5. Ex-4 Upregulates SIRT1 and Inhibits the Notch Signaling Pathway in Senescent PDLSCs

**Figure 7:**
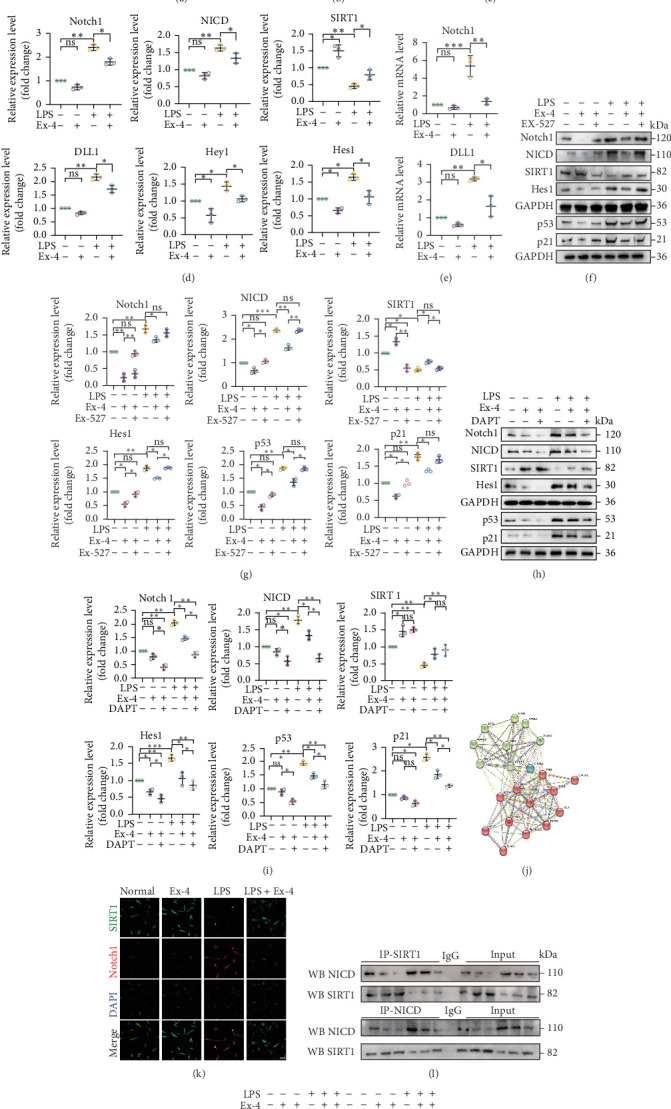
Ex-4 inhibits Notch1 signaling pathway by upregulating SIRT1 expression. (A, B) Immunofluorescence staining expression of Notch1, Hes1 in cells of normal, Ex-4, LPS, and LPS + Ex-4 groups. Scale bar = 100 μm. (C, D) Western Blot detected the expression levels of Notch signaling pathway-related proteins (Notch1, NICD, SIRT1, DLL1, Hey1 and Hes1) and SIRT1 in the cells of normal, Ex-4, LPS, and LPS + Ex-4 groups. (E) The expression levels of Notch1 and DLL1 were assessed by qRT-PCR in cells of normal, Ex-4, LPS, and LPS + Ex-4 groups. (F, G) SIRT1 inhibitors were added, and the experiments were grouped into the following groups: the normal, the Ex-4, the Ex-4 + EX-527, the LPS, the LPS + Ex-4, and the LPS + Ex-4 + EX-527 groups. The expression levels of SIRT1, Notch signaling pathway-related proteins (Notch1, NICD, and Hes1) and senescence-related proteins (p53 and p21) were detected by western blot. (H, I) Notch pathway inhibitors were added, and the experiments were grouped into the following groups: the normal, the Ex-4, the Ex-4 + DAPT, the LPS, the LPS + Ex-4, and the LPS + Ex-4 + DAPT groups. The expression levels of SIRT1, Notch signaling pathway-related proteins (Notch1, NICD, and Hes1), and senescence-related proteins (p53 and p21) were detected by western blot. (J) Interaction graph of Notch1 with SIRT1. (K) Immunofluorescence double staining of Notch1 and SIRT1 in cells of normal, Ex-4, LPS, and LPS + Ex-4 groups. Scale bar = 100 μm. (L) Co-IP analysis of NICD and SIRT1 interactions. (M) Detection of NICD acetylation levels by IP and protein blotting using antiacetylation antibodies. Data are presented as mean ± standard deviation (*n* = 3 independent observations). *⁣*^*∗*^*p* < 0.05; *⁣*^*∗∗*^*p* < 0.01; *⁣*^*∗∗∗*^*p* < 0.001; ns, not significant.

The Notch pathway is closely related to cell growth and development [39]. As shown by immunofluorescence (Figure 7A,B), western blot analysis (Figure 7C,D), and qRT-PCR (Figure 7E), the Notch pathway played a role in inflammatory microenvironment-induced senescence of PDLSCs. In senescent PDLSCs, the expression levels of DLL1, Notch1, NICD, Hey1, and Hes1 were significantly increased, and the expression level of SIRT1 was decreased. The expression levels of DLL1, Notch1, NICD, Hey1, and Hes1 were decreased, and the expression levels of SIRT1 were increased after Ex-4 treatment.

Studies have shown that the Notch signaling pathway is a downstream effector pathway of SIRT1 [37, 40]. Therefore, we next explored whether Ex-4 can affect the expression level of Notch signaling pathway proteins by regulating SIRT1. We added SIRT1 inhibitor (EX-527) and Notch pathway gamma-secretase inhibitors (DAPT). WB results (Figure 7F,G) showed that when SIRT1 was inhibited, the expression of Notch signaling pathway-related proteins (Notch1, NICD, and Hes1) and aging-related proteins (p53 and p21) in the LPS + Ex-4 + EX-527 group increased compared with the LPS + Ex-4 group. Compared with the Ex-4 group, the expression of Notch signaling pathway-related proteins (Notch1, NICD, Hes1) and aging-related proteins (p53 and p21) in the Ex-4 + EX-527 group increased. The results showed that inhibition of SIRT1 could reverse the antiaging effect mediated by Ex-4. After DAPT was added, WB results (Figure 7H,I) showed that when Notch1 was inhibited, compared with the LPS + Ex-4 group, there was no significant difference in the expression of SIRT1 in the LPS + Ex-4 + DAPT group, and the expression of Notch signaling pathway-related proteins (Notch1, NICD, Hes1) and aging-related proteins (p53 and p21) decreased. Compared with the Ex-4 group, there was no significant difference in the expression of SIRT1 in the Ex-4 + DAPT group, and the expression of Notch signaling pathway-related proteins (Notch1, NICD, and Hes1) and aging-related proteins (p53 and p21) decreased. Based on the above results, we conclude that Ex-4 delays PDLSCs' senescence induced by an inflammatory microenvironment through the SIRT1/Notch1 pathway.

### 3.6. Interaction of SIRT1 With Notch1

Studies have shown that SIRT1 can mediate the deacetylation of NICD and cause its degradation, playing a protective role in solid organ damage [41]. Therefore, we next explored whether Ex-4 can affect the expression level of NICD by regulating SIRT1. Through the original letter analysis, we found an interaction between SIRT1 and Notch1 (Figure 7J). Results of immunofluorescence staining (Figure 7K) showed that Notch1 and SIRT1 were coexpressed in PDLSCs.

Co-IP experiments (Figure 7L) demonstrated that, compared with the normal group, LPS stimulation significantly enhanced the interaction between NICD and SIRT1, indicating increased intracellular Notch1–SIRT1 binding within the inflammatory microenvironment. This finding is consistent with previous reports and further confirms the functional interrelationship between SIRT1 and the Notch1 signaling pathway [39]. In comparison to the Ex-4 group, treatment with the γ-secretase inhibitor DAPT did not significantly alter SIRT1 protein levels. Furthermore, we observed that reduced SIRT1 expression resulted in the accumulation of NICD, which in turn promoted cell apoptosis and senescence. Additional Co-IP assays (Figure 7M) revealed that under inflammatory serum stimulation, the acetylation level of NICD increased in PDLSCs, suggesting either impaired deacetylase activity or enhanced acetyltransferase activity under these conditions. Notably, Ex-4 treatment markedly attenuated LPS-induced NICD acetylation, implying that Ex-4 may facilitate SIRT1-mediated deacetylation or inhibit acetyltransferase activity targeting NICD. In summary, inflammation enhances the NICD–SIRT1 interaction and modulates the acetylation status of NICD, while Ex-4 regulates this axis by promoting NICD deacetylation, potentially through enhancing SIRT1 activity or strengthening its association with NICD.

## 4. Discussion

Many investigations have evaluated treatments for periodontitis, with traditional methods primarily focused on plaque removal. However, even with these methods, missing periodontal tissue cannot be restored [42, 43]. PDLSCs have a high differentiation capacity and are seed cells for tissue engineering. However, senescent PDLSCs have a significantly reduced capacity to differentiate into osteoblasts, which hinders tissue repair [43, 44]. Therefore, measures that counteract the senescence of PDLSCs and reduce SASP-associated cellular damage would benefit the clinical application of these stem cells for the treatment of periodontitis. In this study, we found that Ex-4 retarded the senescence of PDLSCs in an inflammatory microenvironment. The results provided the basis for a new approach and direction for future research into periodontal therapy.

Inflammation is intricately connected to cellular senescence. Previous studies have shown that cellular senescence is induced by an inflammatory microenvironment, leading to the impairment of key cellular functions [45–48]. As one of the main components of the cell wall of Gram-negative bacteria, LPS is an important stimulator of the innate immune response and is considered the main pathogenic factor of periodontal disease, resulting in loss of periodontal tissue and eventual tooth loss. In recent years, LPS has been used to simulate the inflammatory microenvironment as a common modeling method. Studies have shown that in the inflammatory environment induced by LPS (10 μg/mL), the osteogenic differentiation of PDLSCs is significantly weakened, with a significant decrease in ALP activity [49, 50]. Lan et al. [51] demonstrated that 10 μg/mL *P. gingivalis* LPS inhibited the proliferation of PDLSCs. LPS directly or indirectly inhibited the proliferation of PDLSCs through LPS-induced inflammatory cytokines. Our previous studies showed that when the concentration of LPS was 10 and 100 μg/mL, the proliferation of PDLSCs increased on the third day. On the 7th day of measurement, proliferation was lower than that of the blank control group, and the osteogenic ability of PDLSCs was inhibited [16]. Previous studies have demonstrated successful induction of cellular senescence by treating cells with various concentrations of LPS for different time periods as a means by which to model senescence [52–55]. The outcomes of the current study revealed that cellular senescence was induced in cells treated with 10 µg/mL LPS for 6 days, without apoptosis. Therefore, in the present study, PDLSCs were cultured in medium containing 10 µg/mL LPS to mimic the inflammatory microenvironment at the periodontal tissue site. Sequencing results demonstrated that the inflammatory environment induced by LPS produced senescence in PDLSCs. Compared with PDLSCs in normal medium, SA-β-gal-positive cells were increased, the cell cycle was arrested in the G0/G1 phase, and the expression of senescence-related proteins was significantly increased after sustained LPS induction. Moreover, the mitochondrial function of senescent PDLSCs was significantly altered, and the level of oxidative stress increased. The proliferative and migratory capacities of senescent PDLSCs were also affected. Therefore, we conclude that the inflammatory environment induces senescence in PDLSCs.

PDLSCs play an important role in maintaining periodontal tissue health and alveolar bone dynamic homeostasis. When PDLSCs senesce, their differentiation capacity is greatly reduced, leading to bone tissue destruction [55, 56]. In this study, we examined the osteogenic capacity of senescent PDLSCs by WB assays and alizarin red staining. The results showed that the osteogenic capacity of senescent PDLSCs was significantly decreased. Ex-4 is a full agonist of the GLP-1 receptor, which produces insulin action. Recent studies have found that Ex-4 has a protective effect on cells and can delay cellular senescence. Ex-4 not only promotes osteogenic differentiation, but also prevents apoptosis and senescence [17, 57, 58]. In the present study, we found that SA-β-gal-positive cells were reduced after Ex-4 treatment compared with the LPS group. We found that the expression of senescence-associated proteins (p53, p-p53, p21, and p16) and DNA damage protein (p-H2A.X) was decreased in PDLSCs after EX-4 treatment as judged by WB assays or qRT-PCR. We also found decreased levels of cellular oxidative stress, improved mitochondrial changes, and enhanced proliferative and migratory capacities. In terms of osteogenesis, both in vivo and in vitro the osteogenic capacity of senescent PDLSCs was significantly reduced, which was partially restored after Ex-4 treatment. In the current study, Ex-4 retarded the senescence of PDLSCs in the inflammatory microenvironment, as evidenced by cell differentiation and proliferation capacity, SA-β-gal staining, and RNA and protein levels of relevant senescence genes. This finding is consistent with a previous study [16], which demonstrated that metformin prevented oxidative stress-induced senescence, thereby protecting the osteogenic differentiation of PDLSCs. Therefore, in the present study, we concluded that Ex-4 effectively delayed the senescence of PDLSCs.

The Notch pathway plays a vital role in cell growth and development [59, 60] including tooth development and regeneration of damaged tissues related to mature teeth [61, 62]. The pathway has also been linked to osteogenic differentiation of PDLSCs [63]. The Notch pathway's upregulation through LPS has been demonstrated [64]. The relationship between Notch signaling and cellular senescence has been demonstrated in various senescence models. In endothelial cells, upregulation of Notch1 is associated with cellular senescence [65, 66]. Notch is also involved in triggering liver sinusoidal endothelial cells (LSECs) senescence and senescence-associated secretory phenotypes, which disrupt liver regeneration. Blocking Notch with a γ-secretase inhibitor (DAPT) reduces senescence and promotes LSEC amplification [67]. Notch has been implicated in replicative senescence of PDLSCs and is controlled by Vc treatment [9]. Although it is established that Vc can delay the replicative senescence of dental stem cells by regulation of Notch3, the role of Notch1 in the senescence of inflammatory microenvironment PDLSCs is ambiguous. In this study, we found that the expression levels of Notch signaling pathway-related proteins (DLL1, Notch1, NICD, Hes1, and Hey1) were increased in senescent PDLSCs induced by an inflammatory microenvironment. Notably, we demonstrated that Ex-4 partially eliminated the promoting effect of LPS on Notch signal transduction-related proteins. In addition, the expression of related senescence proteins (p53, p-p53, p21, p16, and p-H2A.X) was decreased after Ex-4 treatment. The results showed that Ex-4 could inhibit the activation of the Notch signaling pathway.

It has been reported that Notch1 signal transduction is a downstream effector pathway of SIRT1, which can affect the progression of liver fibrosis, acute lung injury, and osteoporosis [68–70]. SIRT1 catalyzes the deacetylation of histones and a variety of nonhistones, including Notch [71]. SIRT1, as an intrinsic negative regulator of the endothelial Notch signaling pathway, reduces NICD transcription and stability by deacetylation [72]. Kleisi, Chiara et al. found that SIRT1 regulates cardiomyocyte proliferation by deacetylating the intracellular domain of Notch1 (NICD) [73]. In recent years, a large number of studies have shown that the SIRT1/Notch1 signaling pathway is closely related to apoptosis, inflammation, and oxidative stress. Small molecule compounds that block the SIRT1/NICD signaling pathway can prevent organ damage [74, 75]. In this study, we found that the expression of SIRT1 in senescent PDLSCs was significantly decreased, while Ex-4 treatment increased the protein level of SIRT1. This suggests that Ex-4 may induce the production of SIRT1. Based on inhibition of the Notch signaling pathway after Ex-4 treatment, we believe that Ex-4 may affect the senescence of PDLSCs through the SIRT1/Notch1 signaling pathway. Next, we explored the relationship between SIRT1 and Notch1. We found that after adding EX-527, compared with the treatment group, the expression of Notch signaling pathway-related proteins and aging-related factors increased. After adding DAPT, there was no significant difference in the expression of SIRT1 compared with the treatment group, which was consistent with previously published results [37, 69]. Our results indicate that Notch1 signal transduction is a downstream effector pathway of SIRT1. Through Co-IP experiments and NICD acetylation experiments, we believe that Ex-4 may delay the aging process of PDLSCs by deacetylating the intracellular domain of Notch1 by SIRT1. It is certain that in addition to the SIRT1/Notch1 pathway, there are many signaling pathways involved in the aging progression of PDLSCs, such as KEAP1-NRF2 [74], FBLN5/p38 MAPK [75], and CCR7/NF-κB [76]. However, whether Ex-4 can improve the aging of PDLSCs through these pathways remains to be determined. In this study, there are limitations to the in vivo experiments. Osteogenic and proliferative ability of PDLSCs was only assessed in nude mice, which does not adequately verify the role of Ex-4 in vivo. In future studies, we will consider an experimental rat periodontitis model using LPS or ligature to study the effect of Ex-4 on PDLSCs and periodontal ligament tissue damage. In this manner, the effect of Ex-4 in vivo will be further assessed. In the mechanism research [76, 77], although Ex-4 is a known GLP-1 agonist, this study has not yet demonstrated whether the regulatory effect of Ex-4 on SIRT1 is related to the GLP-1 receptor. Future experiments should verify whether the knockout or drug inhibition of GLP-1 can block the effect of Ex-4 on SIRT1.

In this study, we found that the inflammatory microenvironment accelerated the premature senescence of PDLSCs, while Ex-4 delayed the senescence of PDLSCs. We found that Ex-4 exerts its protective effect through the SIRT1/Notch1 pathway. Specifically, Ex-4 promotes the degradation of the NICD protein by activating SIRT1 in PDLSCs, thus exerting its antiaging effect. We hope that our research can provide new targets and perspectives for the analysis and treatment of periodontitis.

## Data Availability

The data used to support the findings of this study are available from the corresponding author upon request.

## Supporting Information

Supporting Information 1 Table S1: Antibodies used in this study.

Supporting Information 2 Table S2: mRNA primers used for qRT-PCR.
