# Supplementary material for: Exendin-4 Reduces Senescence of Inflammation-Induced Periodontal Ligament Stem Cells Through SIRT1/Notch1 Signaling
Source: Stem Cells Int. 2025 Nov 24;2025:7639451. doi: 10.1155/sci/7639451 (PMC12668836; doi:10.1155/sci/7639451)
Supplement: Supporting Information 1 — Table S1: Antibodies used in this study. [file 7639451.f1.docx]

**Supplementary Table 1: Antibodies used in this study.**

| Antibodies | Source | Dilution |
| --- | --- | --- |
| CD146 | Proteintech Group Cat# 17564-1-AP | 1:200 (IF) |
| STRO-1 | Proteintech Group Cat# PE-65184 | 1:200 (IF) |
| p53 | Proteintech Group Cat# 10442-1-AP | 1:1000 (WB) |
| p-p53 | Proteintech Group Cat# 28961-1-AP | 1:1000 (WB) |
| p21 | Proteintech Group Cat# 10355-1-AP | 1:1000 (WB)  1:200 (IHC) |
| p16 | Proteintech Group Cat# 10883-1-AP | 1:1000 (WB) |
| Notch1 | Proteintech Group Cat# 10062-2-AP | 1:1000 (WB)  1:200 (IF) |
| Hes1 | Proteintech Group Cat# Ag26336 | 1:1000 (WB)  1:200 (IF) |
| PCNA | Proteintech Group Cat# 10205-2-AP | 1:1000 (WB) |
| GAPDH | Proteintech Group Cat# 10494-1-AP | 1:1000 (WB) |
| p-H2A.X | Abcam ab131382 | 1:1000 (WB) |
| RUNX2 | Abcam ab92336 | 1:1000 (WB)  1:200 (IF) |
| Ki67 | Abcam ab245113 | 1:200 (IF) |
| NICD | ZenBio Biosciences Cat# 340268 | 1:1000 (WB) |
| Hey1 | Huabio Biosciences Cat# ER62471 | 1:1000 (WB) |
| SIRT1 | Huabio Biosciences Cat# ET1603-3 | 1:1000 (WB) |
|  |  | 1:200 (IF) |
| DLL1 | Huabio Biosciences Cat# ER61747 | 1:1000 (WB) |
| Osteoprotegerin（OPG） | Huabio Biosciences Cat# R1608-4 | 1:200 (IHC) |
| Acetyl-Lysine | Affinity Biosciences Cat# DF7729 | 1:1000 (WB) |
| STRO-1 | Affinity Biosciences Cat# PE-65184 | 1:200 (IF) |
| Dylight 594 Goat Anti-Mouse | Abbkine Cat# A23410 | 1:200 (IF) |
| Dylight 649 Goat Anti-Rabbit | Abbkine Cat# A23620 | 1:200 (IF) |
| Dylight 488 Goat Anti-Rabbit | Abbkine Cat# A23220 | 1:200 (IF) |
| IgG-HRP | ZSGB-BI0 Cat# SP-9002 | 1:200 (IF) |
