# Supplementary material for: Exendin-4 Reduces Senescence of Inflammation-Induced Periodontal Ligament Stem Cells Through SIRT1/Notch1 Signaling
Source: Stem Cells Int. 2025 Nov 24;2025:7639451. doi: 10.1155/sci/7639451 (PMC12668836; doi:10.1155/sci/7639451)
Supplement: Supporting Information 2 — Table S2: mRNA primers used for qRT-PCR. [file 7639451.f2.docx]

**Supplementary Table 2: mRNA primers used for qRT-PCR.**

| mRNA | Sequences |
| --- | --- |
| p21 | Forward (5′–3′): TGAATACCGTGGGTGTCAAAGCA |
|  | Reverse (5′–3′): AGACAGGGAGGGAGCCACAATAC |
| p53 | Forward (5′–3′): CCTCAGCATCTTATCCGAGTGG |
|  | Reverse (5′–3′): TGGATGGTGGTACAGTCAGAGC |
| DLL1 | Forward (5′–3′): TGCCTGGATGTGATGAGCAGCA |
|  | Reverse (5′–3′): ACAGCCTGGATAGCGGATACAC |
| Notch1 | Forward (5′–3′): CAGCAAG-AAGAAGCGGAGAG |
|  | Reverse (5′–3′): GAGCACCATCTGAGGCATTC |
| GAPDH | Reverse (5′–3′): TGGATGGTGGTACAGTCAGAGC |
|  | Reverse (5′–3′): CCCTGTTGCTGTAGCCAAAT |
